# Relevance of Intermittent Rivers and Streams in Agricultural Landscape and Their Impact on Provided Ecosystem Services—A Mediterranean Case Study

**DOI:** 10.3390/ijerph16152693

**Published:** 2019-07-28

**Authors:** Tatiana Kaletová, Luis Loures, Rui Alexandre Castanho, Elena Aydin, José Telo da Gama, Ana Loures, Amélie Truchy

**Affiliations:** 1 Faculty of Horticulture and Landscape Engineering, Slovak University of Agriculture in Nitra, 94976 Nitra, Slovakia; 2 Polytechnic Institute of Portalegre (IPP), 7300 Portalegre, Portugal; 3 VALORIZA—Research Centre for Endogenous Resource Valorization, 7300 Portalegre, Portugal; 4 Research Centre for Tourism, Sustainability and Well-being (CinTurs), University of Algarve, 8005-139 Faro, Portugal; 5 Institute of Research on Territorial Governance and Inter-Organizational Cooperation, 41-300 Katowice, Poland; 6 Environmental Resources Analysis Research Group (ARAM), University of Extremadura, 06006 Badajoz, Spain; 7 Faculty of Applied Sciences, University of Dąbrowa Górnicza, 41-300 Katowice, Poland; 8 Departamento de Edafologia, UNEX—Universidad da Extremadura, 06006 Badajoz, Spain; 9 Department of Aquatic Sciences and Assessment, Swedish University of Agricultural Sciences, SE-75007 Uppsala, Sweden

**Keywords:** agricultural landscapes, temporal rivers and streams, ecosystem services, Caia River, Iberian territories

## Abstract

Ecosystem services (ES), as an interconnection of the landscape mosaic pieces, along with temporal rivers (IRES) are an object of research for environmental planners and ecological economists, among other specialists. This study presents (i) a review on the importance of IRES and the services they can provide to agricultural landscapes; (ii) a classification tool to assess the impact of IRES to provide ES by agricultural landscapes; (iii) the application of the proposed classification to the Caia River in order to identify the importance of this intermittent river for its surrounding agricultural landscape. The classification of the ES follows the Common International Classification of Ecosystem (CICES) classification that was adapted for the purposes of this study. Firstly, the list of ES provided by agricultural landscape was elaborated. In the next step, we assessed the potential of IRES to provide ES. Next, IRES impacts to ES within the agricultural landscape were evaluated according to observations from the conducted field monitoring in the study area. This study focuses on the relevance of the intermittent Caia River—a transboundary river in Spain and Portugal—and its ephemeral tributaries in the agricultural landscape. Our study estimates that each hydrological phase of IRES increases the ES provided by the agricultural landscape. However, the dry phase can potentially have negative impacts on several services. The intensification of the agricultural sector is the main provision of the water resource within the Caia River basin, but we were able to identify several other ES that were positively impacted. The present study is in line with the conclusions of other authors who state that IRES constitute a valuable resource which should not be underestimated by society.

## 1. Introduction

Landscapes are mosaics formed by different land use elements interacting among each other. Each element of the mosaic provides several benefits and stressors, services and disservices to the whole ecosystem [1,2,3]. Most of the literature studying land use mosaics has focused on getting detailed information about a given sampling patch [1]. This is a strong shortcoming as these studies typically fail to consider the ecology of the landscape as they omit the inherent inter-connectivity between landscape elements. For instance, even the most utilized land use/land cover classifications can overlook small, but important, pieces within the landscape such as urban gardens, forest patches, hedgerows, and riparian vegetation of permanent or temporal river/streams, as well as the unused land around pivot irrigation soil or alley cropping [4,5,6].

The general definition of Ecosystem Services (ES), provided by the Millennium Ecosystem Assessment (MEA), is “the benefits people obtain from ecosystems” with different classifications dividing them up in provisioning, regulating, supporting and cultural categories [7], or provisioning, regulation and cultural categories—according to the Common International Classification of Ecosystem Services (CICES) [8]. The flow of ES related to agriculture depends on services provided by neighboring ecosystems such as the wildlife habitat, regional kind of food production, forests, aquaculture [3] or anthropic providence [9,10], with several authors [11,12,13] stating that a different spatial and temporal scale of contrasting ecosystems impacts ES. Landscape structure and its spatial arrangements, geographic position and context, the complex interactions between land cover, management and other anthropogenic modifications of the landscape, topography, geology and climate ultimately influence the delivery of general and water-related ES [14,15,16,17,18].

A significant driver of ES change is the change in land use [19]. Water abstraction for irrigation, drinking, industrial or energy purposes can change a perennial river into an intermittent river [20] while, the drainage of agricultural lands or the outflow from a wastewater plant may stabilize the flow of an intermittent stream and temporally change it into a perennial stream [20,21,22]. Moreover, water availability is also influenced by the conversion of natural vegetation to agricultural land [13,23], specifically during summer months (July, August, and September) [24]. Therefore, spatial mapping of the landscape and the interactions occurring at the basin scale is crucial to define the key drivers and pressures of ES change, with their different gradients and variations [25], especially when dealing with limited resources as in agricultural landscape and temporal rivers [26].

Several Mediterranean basins have experienced water shortages in recent decades and future climate change projections foresee further pressure on water resources [27] as this is one of the regions where intermittent rivers and ephemeral streams (IRES) are the predominant type of stream ecosystem [28]. Temporary watercourses, including IRES, are defined as: “waterways that cease to flow at some point in space and time along with their course” [20]. In practice, this means that surface water connectivity is intermittent, i.e., water may be present or absent and, pools may occur resulting in a mosaic of lotic, lentic and terrestrial habitats [22]. Recently, the flow regime of IRES has been proven to vary in duration, time, predictability, volume and spatial extent of flow cessation [28]. Drying events with the intermittent flow in the agricultural landscape usually occur in the short-term (e.g., rill flow—up to several days) or long-term (e.g., flow in drainage/irrigation canals or natural watercourses—from several days up to several months). During the flowing phase, IRES provide the same ES as perennial rivers and streams but, during the pool and especially in the dry phase, the ES can be compared to those provided by the natural, or non-intensive, agricultural landscapes in the Mediterranean basin area where the dry riverbeds can act as a corridor for biotic migrations within the landscape, preferably by small mammals, and where pools may function as their water source [29,30,31]. These habitats differ from the riparian and other terrestrial habitats with different vegetation cover, inundation frequency or biota [30].

Agriculture provides, as well depends on, ecosystem services (e.g., crop production depends on support services such as nutrient and water cycling, pest regulation, soil quality and biodiversity maintenance, etc.), uses 70% of the fresh water worldwide and is practiced on 40% of the Earth’s land surface and in almost 50% of the European Union territory [3,25,32]. Population, not efficient water management, and high demand on the water for different purposes brings the issue to the stakeholders to manage the landscape, which consists of a high proportion of agricultural land, properly. Population growth, as well as the number of undernourished people is still an increasing trend [33]. Therefore, the food production also has to be managed in areas with low water resources. One of those areas is a basin of intermittent rivers and ephemeral streams.

The qualitative and quantitative assessment of ES is usually based on indicators [34] that can be quantified for each landscape element (e.g., [25]) ES. Because indicators are either derived from large-scaled biophysical and socio-economic data or modelled data [25], clear methodologies for assessing ES delivered by IRES are not published yet. In many countries, IRES are overlooked and their significance is neglected. Floods caused by IRES were used for food production in the past, and those techniques are still used in some countries [35]. Literature interlinking IRES and landscape, including agriculture, is rare. Usually the literature focuses on the biota (e.g., [30,35]) or presents the ES overview of IRES in general (e.g., [28]).

Considering the increasing pressure on agriculture to sustain food production for the expanding human population under changing climate conditions, new areas suitable for food production will be sought. We assume that the increase of air temperature will influence the hydrologic balance of currently flowing perennial rivers in arid and semi-arid areas that can result in the rise of IRES in the landscape. Thus, greater attention should be paid to this landscape feature and their potential to provide ES should be further studied. Being aware of this research gap, this study presents (i) a review on the importance of IRES and the services they can provide to agricultural landscapes; (ii) a classification tool to assess the impact of IRES to provide ES by agricultural landscape; and this study (iii) applies the classification in order to identify the importance of the intermittent Caia River for its surrounding agricultural landscape.

## 2. Materials and Methods

### 2.1. Classification of IRES ES and Their Impact

The classification of the ES follows the CICES [8] classification that was adapted for the purposes of this study. Firstly, the list of ES provided by agricultural landscapes was elaborated. In the next step, we assessed the potential of IRES to provide ES from the elaborated list according to [28,36].

Next, IRES impacts to ES within the agricultural landscape were evaluated according to observations from the conducted field monitoring in the study area, which included expertise and evaluation assessment from external professional companies and local agronomists. The impact classification was evaluated throughout a 5-point scale—very positive (++), positive (+), neutral (+/−), negative (−) and not applicable (n/a). The very positive effect meant a significant impact of the ES provided for the agricultural landscape; in the case of a positive effect, the impact was assigned making no distinction among the land use to which the ES was related (IRES or agricultural land). The negative impact was identified for the ES which could be negatively impacted by IRES or lost. The neutral effect expressed the spatial (within basin) or seasonal variability of the impact; and the not applicable effect was assigned in the case of an inability to identify the impact.

### 2.2. Case Study Area

**Figure 1:**
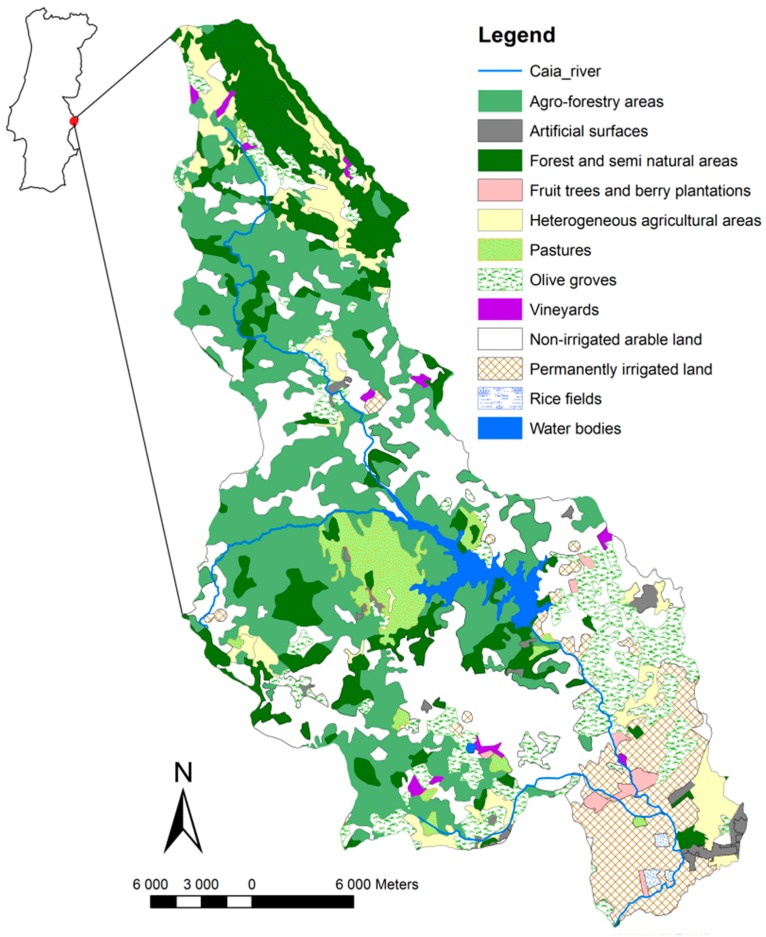
Caia River basin and land use according to the coordination of information on the environment (CORINE) programme land cover project in 2012.

The Caia River is a transboundary, shared water resource; its river basin is approximately 850 km^2^ and it is located between Portugal and Spain (Figure 1). It originates in the Natural Park of the São Mamede ridge, Portalegre—Portugal—and crosses the border to the Spanish territory downstream [37]. The Caia River is a tributary to the Guadiana River near the Spanish city of Badajoz. Yearly average precipitations in the area are 500–600 mm and the potential evapotranspiration is approximately 800–850 mm [37]. The riparian vegetation is very well developed downstream but not upstream where the Caia River is deprived of this kind of vegetation or is only shortly covered within its banks. The irrigated area is situated mainly downstream of the Caia dam. The upstream area is mostly used as non-irrigated arable land and agroforestry (Figure 1), with over 80% of the area dedicated to agroforestry and non-irrigated crops (Table 1). The rest of the area is occupied by artificial surfaces (e.g., buildings, roads), forest, semi-natural areas and water bodies.

The Caia dam divides the river in two parts with different hydrological conditions. The upstream part of the river, from the spring well to the dam, has intermittent river characteristics, while the downstream part is affected by water accumulation in the reservoir and, therefore, has the characteristics of a perennial river. We assume that, without the dam, the whole river would be intermittent. The total capacity of the Caia dam is approximately 203 million m3 [38] and the stored water is used mainly for domestic and irrigation purposes and a minor proportion of the water is used for industry. In the period of 1990–1995, an average of 91.68% of the water was being used for irrigation purposes [39], but during the dry seasons, a higher percentage was being used for domestic use. The Caia River presents ecological, as well as social and economic, benefits, representing an important resource for the region [40]. Field observations in the river basin were conducted in 2018, where we focused on an agricultural landscape survey and detailed mapping of riparian vegetation.

## 3. Results

### 3.1. Interactions of IRES with the Agricultural Landscape

In general, we recognized very positive effects of 29 ES in the case of the flowing phase, 12 in the case of pools and 14 in the case of the dry phase (Table 2). Positive effects are recorded mainly for pool (25) and dry (11) phases. We expected three ES to be affected negatively during the dry phase as they were conditioned to the presence of water. Several ES (12 in total and eight in the dry phase) are identified without an applicable effect of the possibility to be affected by IRES in the agricultural landscape. Those ES (e.g., surface water for drinking or non-drinking purposes) cannot be supported because of the missing main driver of ES (water). Certainly, every area of interest is specific and thus the impact of IRES can vary significantly.

**Figure 2:**
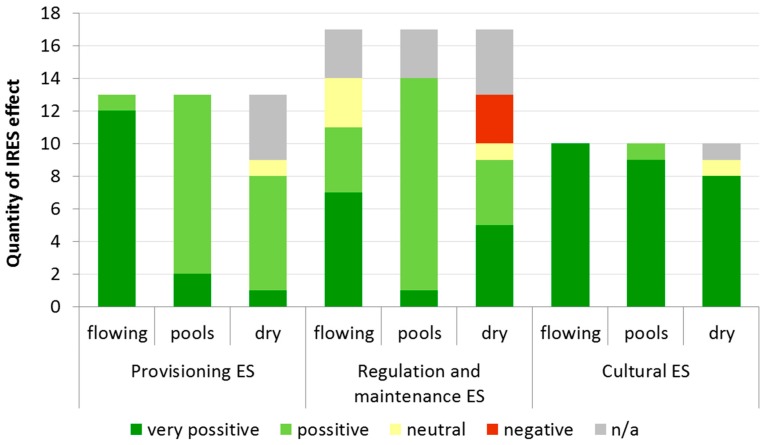
Quantity of IRES effects within the groups of ES.

In general, provisioning ES can be affected by IRES positively or very positively (Figure 2). We have not recognized any negative effect of IRES to agriculture within provisioning ES. During the flowing phase, we identified that 92% of ES which can be provided to agriculture, are very positively affected by IRES. Still, this impact reduces significantly during the pool and the dry phases, to 15% and 7%, respectively.

Several ES (18%) within the group of regulation and maintenance ES can by affected negatively and, for the same amount of ES, it is not possible to identify the potential impact. Hydrological cycle and water flow maintenance, Flood protection and storm protection are ES provided by both agricultural landscape and IRES. However, their influence by interaction between landscape and IRES very depends on the local conditions. Therefore, we prefer to use the assessment not applicable instead of neutral. The negative effect can occur in the case of the dry phase. The hydrological phase of pools can affect the ES provided by agriculture only positively (76%) or very positively (6%).

The comparison of groups of the ES shows that there is a very positive effect mainly for the cultural ES for all IRES stages. It is very difficult to find any information about the negative effect to provide cultural ES. We assume that the flowing phase of IRES can have only a very positive effect on all ES; the dry phase of pools affects one ES positively and neutrally, respectively. In the case of heritage and culture ES, we were not able to identify the impact of IRES.

### 3.2. Benefits to Agriculture from the Caia River

The temporal Caia River is a resource for the irrigation of arable land, given that the amount of available water for crops and orchards can vary due to the presence of riparian vegetation [2]. The stream is also an important source for both wild and domestic animals throughout the year that use the dry channel to migrate between pools that concentrate most of the available drinking water.

Although the intensive monoculture practices of the region associated with the types of crops grown in the watershed (mainly cropland, orchards, vineyards and olive groves) are well known for reducing biodiversity, carbon stocks and water budgets as a result of maximizing production [13,41,42], some areas surrounding the croplands remained unmaintained. Indeed, some landowners do increase biodiversity by creating habitats for natural predators and pollinators, mostly due to (a) governmental incentives (green payments) such as the common agricultural policy (CAP) greening measures, (b) fees paid by sportsmen for hunting excursions that require game to be present, and (c) leftover areas, e.g., those unreachable by the circular operated pivot irrigation systems. Therefore, it is possible to encounter the endemic Iberian painted frog (*Discoglossus galganoi*), the Iberian midwife toad (*Alytes cisternasii*), the Bosca’s newt (*Lissotriton boscai*), and the Schreiber’s green lizard (*Lacerta schreiberi*) in the *Caia* River basin [43], that are able to thrive in the intermittent river as their different growing stages have different water requirements.

The variety of the river phases—flow, pool and dry—allows for the development of lotic, lentic and terrestrial habitats and affect the migration of terrestrial animals, both wild and domestic, because of insufficient water conditions and nutrition with pools remaining as their only available water source during the drought in the Caia River basin [22,31]. Migrating animals and flowing water support the regulatory ES of seed dispersion. Pools are fed by water tables that, by increasing their storage, provide a habitat for fish during the dry season in the area [44].

The Caia dam is a crucial part of flood protection. It protects not only human properties but also the area itself. On one hand, flooded water can contain important nutrients for agriculture and can increase the soil fertility; but, on the other hand, it can be a source of harmful contents (e.g., heavy metals) originating from activities located upstream and accumulating along the river. Without the dam, the permanently irrigated agriculture could not be as intensive during summertime as it is now. A change in crops grown, with lower water requirements and lower transpiration, would be needed. If not, the yearly yield would be lower or none. The intermittency of the river caused by agricultural activities in the area has not yet been recognized. Water uptakes from the river and the dam are regulated, so downstream impacts remain minor.

### 3.3. Agricultural Stressors to the Caia River

The Caia dam collects water during the winter and spring seasons, when the water flow is higher than the requirements for the delivery the different ES. The collected water is mainly used for human consumption and the irrigation of crops, olive trees, vineyards and orchards. Intermittence within the Caia River occurs naturally upstream of the dam, where unmanaged water withdrawal occurs. However, an insufficient water supply can cause intermittence spreading risk downstream of the dam where most irrigated soils are located. The stressors and pressures on water systems occurring within the Caia River basin comprise (a) water quantity—including flowing water frequency, groundwater abstractions, changes in precipitation, temperature and runoff; (b) water quality—the point and diffuse pollution caused by nutrients, chemicals and heavy metalloids built-up; (c) habitat—hydro-morphological alterations; and (d) biota and biological communities—as invasive species [15].

**Figure 3:**
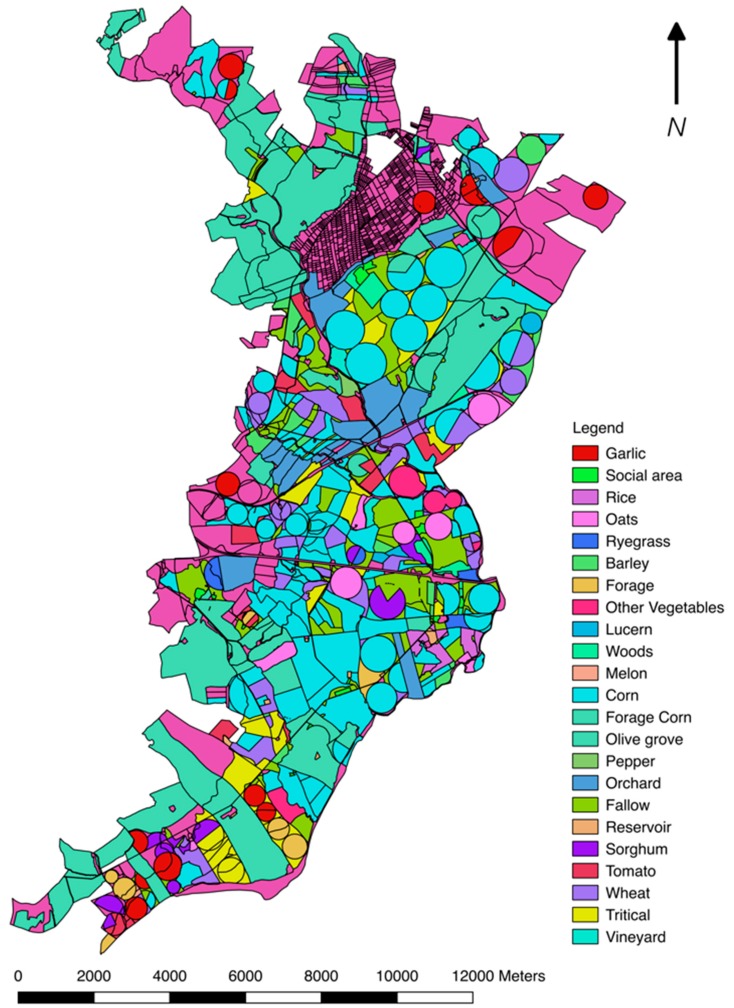
Typical crop distribution (data from 2012).

Because of the edaphic-climatic conditions of the region, a comparison between the amount of water consumed by spring/summer crops and the actual precipitation reveals a great disproportion that affects the remaining available water for other activities in the area. The average precipitations are able to cover from 43 to 92% of crop water demand. Table 3 indicates the water consumption of most irrigated crops from the Caia dam since 1990 [39] until 2016 [45], with the five most water-consuming crops having a combined consumption of 39,351,285 m^3^ year^−1^ of water in 6,493.92 ha. Figure 3 shows the usual grown crops, as well as other relevant information, in the southeastern part of the basin.

A comparison of land use change between the years 1990 and 2012 shows that agricultural areas decreased by −2.67%, and were replaced by forest and semi natural areas (+1.95%) and artificial surfaces (+0.65%). We also recognized change within the agricultural areas, where the main decrease was observed for non-irrigated arable land (−12.41%) and rice fields (−0.85%). On the other hand, there was an increase in areas of olive groves (+3.12%), pastures (+3.64%) and permanently irrigated land (+2.35%), which can be recognized as land use change, leading to higher water consumption and water demand in general.

**Figure 4:**
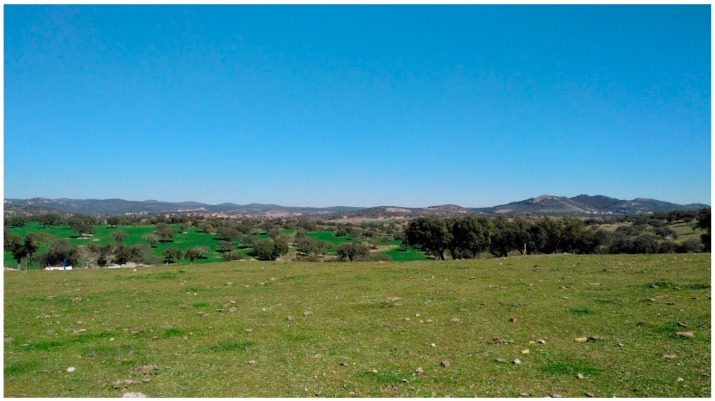
Overgrazing of the agricultural landscape—the river corridor is in the valley (author: Kaletová, 2018).

**Figure 5:**
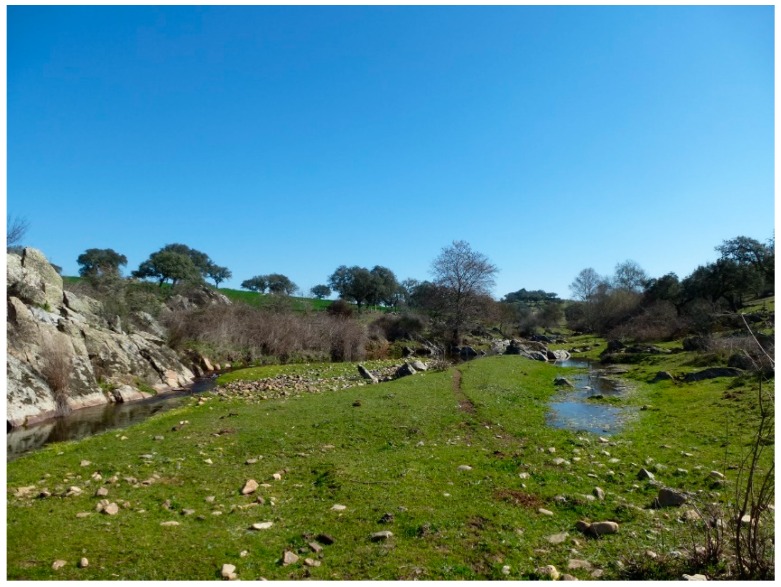
Caia riverbed, with the riparian vegetation within the agricultural landscape (author: Kaletová, 2018).

The mounds within the basin are generally poorly maintained, with signs of overgrazing by the cattle and other animals (Figure 4). Animal breeding and related overgrazing affected the riparian vegetation and created opportunity for invasive species such as *Acacia* spp. (Figure 5).

## 4. Discussion

Interactions between IRES and the agricultural landscape have been extensively described in the literature [46,47,48], as well as the ES they provide [2,12,15,49,50]. However, the possible impacts on the ES remain understudied and, as [26] stated, understanding the impacts of landscape changes on the water supply is crucial for the effective assessment of water-related ES. The dynamic mosaic that characterizes the agricultural landscape [1,51] is subjected to variable changes in structure (see Table 1), crop production, cycles of cultivation and harvest, orchards flowering and fruiting, climate conditions, unstable ecological processes and management practices [1,51,52]. An effort to maximize the provision of one ecosystem service often tends to decline the production of other ES, while the demand for the provision increases [4,7]. Overall, IRES in the landscape tend to have more positive than negative effects (Table 2) with the main positive impact occurring during its flowing phase. Beyond the ES provided by flowing streams as water supplying, fish production, recreational space or aesthetics [53], we identified many others ES listed in Table 2. However, several provisional services can be reduced, or even lost, during the pool or drought phase, which is in accordance with previous studies [28,36]. Pool phases are mainly used by livestock or, at a smaller scale, for irrigation purposes and may bring positive or neutral effects depending on the agricultural landscape.

As aforementioned, the interactions between IRES and the agricultural landscape can have both positive and negative outcomes. We identified 10 potential benefits of the Caia River for the agricultural landscape and seven stressors from the agricultural landscape to the Caia River (Table 4).

The Caia River provides several benefits for agricultural production within the basin and increases the possibilities for agricultural intensification that, in turn, affects river sustainability. Without the dam, the agricultural intensification practiced during spring and summer would be jeopardized. Therefore, a change in the grown crops, with lower water requirements and lower evapotranspiration, would be required at the risk of low—if any—yields. Consequently, the dam allows converting the rainfed agricultural system to the intensive irrigated agriculture [54].

All living individuals, from microbes to megafauna and vegetation, are often the driving force in the ecosystem affecting water attributes [14]. Indeed, upstream vegetation may affect water distribution, by controlling surface runoff and infiltration into the soil, as well as water quality and supply in the landscape—that is invariably linked to precipitation [2,55]. In general, areas with root crops and vineyard or orchards without cover crops generate higher surface runoff than those with cereals or fodder crops [56,57], which have significant impacts against soil erosion (both due to water and wind) and, consequently, the siltation watercourses and reservoirs [58,59]. Sediments that once stored in the river channel may reduce the water flow [60]. There is visible different vegetation cover within the Caia River basin as well as in the riparian vegetation according to the surrounding type of landscape. The vegetation cover of the Caia riverbed itself varies during the year according to the flowing phase.

Dry riverbeds allow for soil accumulation of organic matter and fertile substrates for agriculture, cattle grazing or growing annual and perennial crops and orchards. However, the overgrazing, weed infestation, cropping or livestock trampling (as it was also observed during the field monitoring in study area) are stressors for the dry riverbed [61,62]. Nutrient runoff is reduced during the dry hydrological phase, but the flowing phase increases its risk and, downstream, soil fertilization calculation must take this surplus into account. Additionally, the wind erosion of dry riverbeds may affect soil quality by phytopharmaceutical contamination, direct crop abrasion or pathogen contamination [63,64]. The dry channels, due to the wind drift of soil particles, may have a negative ecological impact but the riparian vegetation, as well as the flowing water and pools, are able to reduce or even mitigate it as the riparian vegetation improves ventilation and transpiration of IRES. Moreover, it may be the source of natural predators for parasites while providing shade and cooler temperatures to wild and domestic animals [5,28,62,65]. The intensive agricultural practices can damage the riparian zone and, therefore, have impacts on supporting services and ultimately on final services such as aesthetic value, pollination, erosion and flood control [62].

Retained clay and organic matter as a result of water and wind erosion in the dry riverbed may decrease the total soil and nutrient loss in the area with the developed vegetation of the riverbed also likely to reduce soil erosion [28]. On the other hand, biomass over-extraction in drylands increases erosion [66] and, therefore, an appropriate management of the cropland is necessary.

Dry channels also play an important role as sinks for floodwater and recharges for alluvial aquifers [36] that, together with the water retention and runoff reduced by vegetation, can increase flood control in the area downstream and improve basin and channel management [67].

Several ground-nesting pollinators use the dry riverbed and riparian vegetation for nesting, which increases crop and orchard pollination. The importance of wild pollinators that fly from nesting sites in nearby habitats is documented in [2].

According to [36], the flowing phase of IRES may affect the efflux of greenhouse gases as the previously dry channels and pools that acted as carbon sinks storing organic matter, now act as sources ultimately affecting climate regulation in the local and regional scales.

Regarding impacts on water quality, treated or untreated wastewater from households, mining or industries can contain a small proportion of nutrients important for the optimal growth of crops. However, heavy metals, bacteria, viruses and other polluting compounds are present as well, with some being harmful for crops or animals. The water quality of the Caia River is mostly affected by untreated wastewater from industries and diffuse pollution from agriculture and animal production, as there are several small companies (e.g., olive oil mills, piggeries and abandoned mines) in the area contributing to the outflow of untreated wastewater into the river [68].

Nutrients in the surface water have a major impact on water quality [19] that, depending on their concentrations, may be positive or negative. While lower amounts of nutrients can be a source of natural food for aquatic animals such as fish, higher amounts can cause eutrophication of pools during summertime, although the flowing water in IRES may transform some nitrate into non-reactive forms [69,70,71]. Because of upstream activities, the water used in downstream agriculture contains higher amounts of salts and heavy metalloids. The Iberian Peninsula rivers, to which the Caia River is a good representative, have strong seasonal fluctuations in their flow regime: (a) dry or reduced to pools during the summer, (b) flooding in the winter and spring and (c) presenting poorer water quality downstream than upstream during the summer [44,72].

Cultural ES are especially well supported by IRES as they increase the area accessibility for anthropic recreational activities such as canyoning, swimming, fishing, and corridors for hikers, bikers, horse riders or motorcyclists. The high recreation value of the Caia River area can be deduced from the fact that 17,664 recreational and 209 commercial fishing licenses were issued within the Portalegre district.

Access of heavy machinery to the area may also be improved. Because of allelopathy and competition for resources such as water, nutrients, light and space weeds are synonymous with lower yields or higher production costs and, thus, usually undesired by landowners. Nevertheless, these weeds are an important piece of biodiversity as they can attract more pollinators and, therefore, increase crop pollination, a fact that was observed in the Caia River.

From the abovementioned, it is possible to recognize, that most of the ES provided by agriculture could be boosted up by the Caia River. The Caia River itself does not provide ES such as plant-based or animal-based resources of energy, cultivated crops, pollination, pest and disease control or heritage, but without the ES assigned to the intermittent part of the Caia River, those ES could not be created or the potential would be very low.

Nonetheless, our work estimated that each hydrological phase of IRES increases the ES provided by the agricultural landscape. The dry phase of IRES has a neutral impact on several ES or is not applicable, at least. Thus, the present study confirms the conclusions of [36], that IRES should not be undervalued by society. In fact, to define the temporal dynamic of ES provided by different hydrological phases of IRES, a similar approach to the temporal dynamics of different ES provided by cover crops described by [12] could be further studied and developed.

## 5. Conclusions

In this study, we focused on the benefits provided by the Caia River to the agricultural landscape and on the stressors from the agricultural landscape to the Caia River that may decrease or increase the ES. We identified 10 potential benefits of the Caia River for the agricultural landscape and seven stressors from the agricultural landscape to the Caia River. During the flowing phase, we recognized that 92% of ES can be provided to the agriculture, which can be very positively affected by IRES; but only 15 and 7% in the case of pools and the dry phase, respectively. The comparison of groups of ES shows that there is a very positive effect, mainly for the cultural ES for all IRES stages.

Our study estimates that each hydrological phase of IRES increases the ES provided by the agricultural landscape. However, the dry phase can potentially have a negative impact on several services. The intensification of the agricultural sector is the main provision of the water resource within the Caia River basin, but we were able to identify several other ES that were positively impacted.

Impacts caused by the Caia River itself and by the smaller, ephemeral, tributaries within the basin should not be excluded from the ecological equation. The present study is in line with the conclusions of other authors that state that IRES should be ecologically and economically valued. Future research may provide relevant insights in order to define the temporal dynamics of ES provided by the different hydrological phases of IRES similarly to the temporal dynamics of the different ES provided by cover crops as published.

## Figures and Tables

**Table 1:** Land use within Caia River basin and structure of agricultural land according to the coordination of information on the environment (CORINE) programme land cover project in 1990 and 2012.

Land Use	1990		2012	
	Area (ha)	Area (%)	Area (ha)	Area (%)
Artificial surfaces	513	0.61%	1061.46	1.26%
Forest and semi natural areas	10,047.79	11.94%	11,695.53	13.89%
Water bodies	1537.01	1.83%	1584	1.88%
Agricultural areas (of wich):	72,079.04	85.63%	69,835.77	82.96%
Non-irrigated arable land	31,747.02	37.71%	21,293.54	25.30%
Permanently irrigated land	4782.29	5.68%	6758.42	8.03%
Rice fields	892.24	1.06%	177.64	0.21%
Vineyards	137.46	0.16%	497.12	0.59%
Fruit trees and berry plantations	553.41	0.66%	573.14	0.68%
Olive groves	5498.86	6.53%	8126.14	9.65%
Pastures	25.97	0.03%	3092.53	3.67%
Heterogeneous agricultural areas	4407.37	5.24%	5264.27	6.25%
Agroforestry areas	24,034.42	28.55%	24,052.97	28.57%

**Table 2:** List of ecosystem services provided by the agricultural landscape; the relation of the hydrological phase of IRES to provide each ES and the impact of the hydrological phase of IRES in the ES by the agricultural landscape.

ES Provided by the Agricultural Landscape	ES Provided by IRES ^(a)^			Impact of IRES ^(b)^		
	Flowing	Pools	Dry	Flowing	Pools	Dry
**Provisioning ES**						
Cultivated crops	1	1	1	++	+	+
Reared animals and their outputs	2	1	1	++	+	+
Wild plants	2	1	1	++	+	+/−
Wild animals and their outputs	2	2	2	+	+	+
Surface water for drinking	2	1	0	++	+	n/a
Ground water for drinking	2	1	1	++	++	n/a
Fibers and other materials from plants, algae and animals for direct use or processing	2	2	2	++	+	+
Materials from plants, algae and animals for agricultural use	2	1	2	++	+	+
Genetic materials from all biota	2	2	2	++	++	++
Surface water for non-drinking purposes	2	1	0	++	+	n/a
Ground water for non-drinking purposes	2	1	1	++	+	n/a
Plant-based resources of energy	1	2	2	++	+	+
Animal-based resources of energy (e.g., from manure)	2	1	1	++	+	+
**Regulation and maintenance ES**						
Filtration/sequestration/storage/accumulation by ecosystems	2	2	2	++	+	−
Mediation of smell/noise, visual impacts	2	1	1	++	+	+
Mass stabilization and control of erosion rates	1	2	2	+/−	+	++
Buffering and attenuation of mass flows (e.g., sediment retention)	1	2	2	+/−	+	++
Hydrological cycle and water flow maintenance	2	1	0	n/a	n/a	n/a
Flood protection	1	1	1	n/a	n/a	n/a
Storm protection	n/a	n/a	n/a	n/a	n/a	n/a
Ventilation and transpiration	2	2	2	+	+	−
Pollination and seed dispersal	1	1	1	++	++	++
Maintaining nursery populations and habitats (e.g., traditional orchard)	2	2	2	++	+	+
Pest control	2	1	1	+	+	++
Disease control	2	2	2	+/−	+	++
Weathering processes (e.g., water and wind erosion)	2	2	2	+	+	+
Decomposition and fixing processes (e.g., gross nitrogen balance)	2	2	2	+	+	n/a
Chemical condition of freshwater	2	1	0	++	+	−
Global climate regulation by reduction of greenhouse gas concentrations (e.g., carbon sequestered by plants)	2	1	0	++	+	+
Micro and regional climate regulation	2	1	1	++	+	+/−
**Cultural ES**						
Experiential use of plants, animals and landscapes in different environmental settings	2	1	1	++	++	++
Physical use of landscapes in different environmental settings	2	2	2	++	++	++
Scientific studies	2	2	2	++	++	++
Education (e.g., didactic farm)	2	2	2	++	++	++
Heritage, culture (e.g., monuments, certified products)	2	2	2	++	+	n/a
Entertainment (contest, competition)	2	2	2	++	++	++
Aesthetic (e.g., photos, visitors)	2	2	2	++	++	++
Symbolic (e.g., trees, species)	2	2	2	++	++	+/−
**Sacred and/or religious (e.g., pilgrim paths, chapels)**	2	2	2	++	++	++
**Existence (protected areas, e.g., Natura 2000, UNESCO)**	2	2	2	++	++	++

(a) Provisioning ES is: 2—sufficient, 1—limited, 0—not sufficient; (b) impact of IRES: ++ very positive, + positive, − negative, +/− neutral, n/a not applicable.

**Table 3:** Average water consumption of the main irrigated crops in the Caia River basin.

Crop	Area (ha)	WC * (m^3^ ha^−1^ year^−1^)	Total WC (m^3^ year^−1^)
Other Vegetables	121.98	500	60,990
Vineyard	61.01	2000	122,020
Oats	228.02	600	136,812
Barley	211.88	800	169,504
Triticale	452.84	1000	452,840
Wheat	628.00	1000	628,000
Sorghum	161.69	5000	808,450
Garlic	287.87	3500	1,007,545
Rice	78.09		1,093,260
Tomato	206.52	6000	1,239,120
Orchards	550.77	6500	3,580,005
Olive grove	2914.66	3000	8,743,980
Corn	2743.88	9000	24,694,920

* average; WC: water consumed.

**Table 4:** Analysis of the benefits of the Caia River for agriculture and stressors from agriculture to the Caia River.

Benefits	Stressors
surface water	intensification and monoculture
ground water	soil water erosion
nutrition for animals	wind erosion
nutrients and organic matter	nutrients
pollinators (wild pollinators from nearby areas)	competition for resources
soil fertility	lack of water
genetic diversity	water quality
natural predators/parasitoids	invasive species
research approach	
soil water–air regime	
